# Health outcomes of 1000 children born to mothers with inflammatory bowel disease in their first 5 years of life

**DOI:** 10.1136/gutjnl-2019-319129

**Published:** 2020-10-12

**Authors:** Shannon Linda Kanis, Sanne Modderman, Johanna C Escher, Nicole Erler, Ruud Beukers, Nanne de Boer, Alexander Bodelier, Annekatrien C T.M Depla, Gerard Dijkstra, Anne-Baue Ruth Margaretha van Dijk, Lennard Gilissen, Frank Hoentjen, Jeroen M Jansen, Johan Kuyvenhoven, Nofel Mahmmod, Rosalie C Mallant-Hent, Andrea E van der Meulen-de Jong, Anahita Noruzi, Bas Oldenburg, Liekele E Oostenbrug, Pieter C.J. Ter Borg, Marieke Pierik, Mariëlle Romberg- Camps, Willem Thijs, Rachel West, Alison de Lima, C Janneke van der Woude, Initiative on Crohns and Colitis (ICC)

**Affiliations:** 1 Gastroenterology and Hepatology, Erasmus Medical Center, Rotterdam, The Netherlands; 2 Pediatric Gastroenterology, Erasmus MC Sophia Children Hospital, Rotterdam, The Netherlands; 3 Biostatistics, Erasmus Medical Center, Rotterdam, The Netherlands; 4 Gastroenterology and Hepatology, Albert Schweitzer Ziekenhuis, Dordrecht, The Netherlands; 5 Gastroenterology and Hepatology, Amsterdam UMC, Amsterdam, The Netherlands; 6 Gastroenterology and Hepatology, Amphia Hospital site Molengracht, Breda, The Netherlands; 7 Gastroenterology and Hepatology, Slotervaart Hospital, Amsterdam, The Netherlands; 8 Gastroenterology and Hepatology, University of Groningen, Groningen, The Netherlands; 9 Gastroenterology and Hepatology, Catharina Ziekenhuis, Eindhoven, The Netherlands; 10 Gastroenterology and Hepatology, Radboudumc, Nijmegen, The Netherlands; 11 Gastroenterology and Hepatology, Onze Lieve Vrouwe Gasthuis, Amsterdam, The Netherlands; 12 Gastroenterology and Hepatology, Spaarne Hospital, Haarlem, The Netherlands; 13 Gastroenterology and Hepatology, Sint Antonius Ziekenhuis, Nieuwegein, The Netherlands; 14 Gastroenterology and Hepatology, Flevo Hospital, Almere, The Netherlands; 15 Gastroenterology and Hepatology, Leiden University Medical Center, Leiden, The Netherlands; 16 Gastroenterology and Hepatology, Utrecht Hospital, Utrecht, The Netherlands; 17 Gastroenterology and Hepatology, Zuyderland Medisch Centrum Heerlen, Heerlen, The Netherlands; 18 Gastroenterology and Hepatology, Ikazia Hospital, Rotterdam, The Netherlands; 19 Gastroenterology and Hepatology, Maastricht Universitair Medisch Centrum+, Maastricht, The Netherlands; 20 Gastroenterology and Hepatology, Zuyderland Medical Centre Sittard-Geleen, Sittard-Geleen, The Netherlands; 21 Gastroenterology and Hepatology, Martini Hospital, Groningen, The Netherlands; 22 Gastroenterology and Hepatology, Franciscus Gasthuis, Rotterdam, New Caledonia

**Keywords:** inflammatory bowel disease, non-steroidal anti-inflammatory drugs, infliximab, azathioprine

## Abstract

**Objective:**

The aim of this study was to describe the long-term health outcomes of children born to mothers with inflammatory bowel disease (IBD) and to assess the impact of maternal IBD medication use on these outcomes.

**Design:**

We performed a multicentre retrospective study in The Netherlands. Women with IBD who gave birth between 1999 and 2018 were enrolled from 20 participating hospitals. Information regarding disease characteristics, medication use, lifestyle, pregnancy outcomes and long-term health outcomes of children was retrieved from mothers and medical charts. After consent of both parents, outcomes until 5 years were also collected from general practitioners. Our primary aim was to assess infection rate and our secondary aims were to assess adverse reactions to vaccinations, growth, autoimmune diseases and malignancies.

**Results:**

We included 1000 children born to 626 mothers (381 (61%) Crohn’s disease, 225 (36%) ulcerative colitis and 20 (3%) IBD unclassified). In total, 196 (20%) had intrauterine exposure to anti-tumour necrosis factor-α (anti-TNF-α) (60 with concomitant thiopurine) and 240 (24%) were exposed to thiopurine monotherapy. The 564 children (56%) not exposed to anti-TNF-α and/or thiopurine served as control group. There was no association between adverse long-term health outcomes and in utero exposure to IBD treatment. We did find an increased rate of intrahepatic cholestasis of pregnancy (ICP) in case thiopurine was used during the pregnancy without affecting birth outcomes and long-term health outcomes of children. All outcomes correspond with the general age-adjusted population.

**Conclusion:**

In our study, we found no association between in utero exposure to anti-TNF-α and/or thiopurine and the long-term outcomes antibiotic-treated infections, severe infections needing hospital admission, adverse reactions to vaccinations, growth failure, autoimmune diseases and malignancies.

**Significance of this study**

**What is already known on this subject?**

- Patients with inflammatory bowel disease (IBD) often need maintenance treatment during pregnancy.
- Anti-TNF-α and immunomodulators both cross the human placenta.

**What are the new findings?**

- In our multicentre retrospective study assessing health outcomes in children born to mothers with IBD with a follow-up of 5 years we found:
  - No association between in utero exposure to anti-TNF-α and/or thiopurine and the outcomes antibiotic-treated infections and severe infections needing hospital admission.
  - No evidence for an association between exposure to anti-TNF-α and/or thiopurine during pregnancy and adverse reactions to vaccination, growth failure, autoimmune diseases and malignancies.
  - An association between thiopurine use during pregnancy and intrahepatic cholestasis of pregnancy without affecting birth outcomes and long-term health outcomes of children.

**How might it impact on clinical practice in the foreseeable future?**

- Anti-TNF-α and thiopurines, as monotherapy or combination treatment, may be used during pregnancy to maintain disease remission.
- Therapeutic drug monitoring during pregnancy should be introduced in the case of thiopurine use to avoid maternal exposure to high levels of 6-methylmercaptopurine (6-MMP).

## Introduction

Inflammatory bowel disease (IBD) represents chronic diseases that may be maintained in remission by different types of immunosuppressive medication and typically affects patients in their reproductive years. Inevitably a part of the female patients will require treatment during pregnancy. Anti-TNF-α) and immunomodulators, as monotherapy or in combination, are used increasingly to maintain disease remission^1 2^ and both types of drugs cross the human placenta.^3–8^ The use of these drugs during pregnancy is not associated with adverse pregnancy outcomes, such as preterm delivery, low birth weight or congenital abnormalities,^9–15^ however, the impact on the development of children’s immune system, growth and risk of autoimmune diseases and malignancies later in life is poorly studied.

Clinical data regarding infection risk in infants exposed to anti-TNF-α and/or thiopurine in utero show conflicting results. An almost threefold increased infection risk was found in a prospective study of infants exposed to the combination of anti-TNF-α and thiopurine compared with infants exposed to anti-TNF-α monotherapy.^16^ However, our study group reported comparable infection rates in infants exposed to anti-TNF-α monotherapy and infants exposed to the combination of anti-TNF-α and thiopurine.^17^ In addition, a retrospective study found no association between anti-TNF-α exposure and severe infections in children needing hospital admission.^18^ Another national registry-based study also found no difference in infections in the first year of life in children exposed to anti-TNF in utero.^19^ Overall, follow-up was mostly 1 year, and studies reported only the single health outcome of infections.

The primary aim of our study was, therefore, to assess the effect of IBD drug exposure in utero on the infection rate until 5 years of age. The secondary aims were to assess the effect of IBD drug exposure on adverse reactions to vaccinations, growth development, the risk of autoimmune diseases and malignancies in the first 5 years of life.

## Methods

### Study design

We conducted a multicentre retrospective study in the Netherlands. All 56 Dutch hospitals with a gastroenterology department were asked to participate. In each participating hospital, women diagnosed with IBD and a reported pregnancy or child in their medical chart were identified during an uniform process and were invited per letter. In this letter, we asked women who gave birth after 1999 and who were diagnosed with IBD prior to their pregnancy to respond. In addition, an advertisement was placed by the national IBD patient organisation (Crohn en Colitis Ulcerosa Vereniging Nederland) to recruit additional patients. Outcomes were retrieved during a telephonic interview with mothers and information was later verified in their medical chart. Long-term health outcomes of children were retrieved during the telephonic interview with mothers and also collected from the general practitioner (GPs) with informed consent of both parents/legal gardians. In the Netherlands, the GPs possess the most accurate data because all residents are registered at a general practice that provides primary care. In addition, GPs are the gatekeepers to hospital and specialist care. Hospital-based medical specialists always provide the GPs with written information about hospital admissions and outpatient evaluations. If GP’s could not provide the requested information we used information provided by mothers.

### Outcomes

The following outcomes were collected: IBD disease characteristics, education level, medication use during pregnancy, life style habits (ie, smoking, folic acid intake), IBD surgery prior to pregnancy, disease activity during pregnancy necessitating medication adjustment, mode of delivery, breast feeding for at least 1 month, obstetrical complications (ie, hyperemesis gravidarum, intrahepatic cholestasis of pregnancy (ICP), in utero growth restriction, hypertension / pre-eclampsia, ‘haemolysis, elevated liver enzyme levels and low platelet levels’ (HELLP), gestational diabetes), birth outcomes (ie, birth weight, gestational age and congenital abnormalities) and long-term health outcomes of children until 5 years of age (ie, infections requiring systemic antibiotic treatment, severe infections necessitating hospitalisation, day care attendance, growth failure, adverse reactions to vaccinations, autoimmune diseases and malignancies).

Antibiotic prescription in children is influenced by seasonal differences, with a peak incidence in the winter.^20^ We, therefore, included information per completed follow-up year.

### Study population

The study population consisted of mothers diagnosed with IBD with their offspring born from January 1999 to June 2018. We compared pregnancy and long-term health outcomes of three treatment groups; (1) women using anti-TNF-α monotherapy, (2) women using a thiopurine monotherapy or (3) women using combination treatment of anti-TNF-α and a thiopurine, with controls consisting of women not using anti-TNF-α nor a thiopurine.

### Statistical analyses

All analyses were performed using IBM SPSS statistics (V.21.0). Descriptive statistics of non-normally distributed continuous data are displayed as medians with IQR. The continuous non-normally distributed data were compared using the nonparametric Mann Whitney U test. Categorical data are shown as absolute numbers with percentages and are compared using Fisher’s exact tests. Tests were performed two tailed and tested at a significance level of 0.05, unless stated differently. To analyse the risk of major congenital abnormalities, preterm birth and small for gestational age (SGA) (multivariable) logistic regression was performed. All models were adjusted for the treatment groups. Additional predictor variables were included in the model if they had a p<0.10 in the univariable model and if the group size of the outcome variable allowed for it (which was only the case for preterm birth). Candidates for the predictor variables were: systemic corticosteroids use during pregnancy, disease activity during pregnancy, IBD type, maternal age, smoking during pregnancy, obstetric complications and endoscopy during the pregnancy. Interaction terms between the variables in the multivariable model were tested and included in the final model if significant (p<0.05).

#### Long-term outcomes were evaluated as follows

To analyse the risk of antibiotic-treated infections and severe infections needing hospital admission (multivariable) negative binomial regression models were created, with the log of follow-up years as the offset. All models were adjusted for the treatment groups. Additional predictor variables were included in the model if they had a p<0.10 in the univariable model. The following predictor variables were assessed: systemic corticosteroids use during pregnancy, ICP, smoking during pregnancy, disease activity during pregnancy, obstetric complication, breast feeding, day care attendance and preterm birth. Interaction terms between the variables in the multivariable model were tested and included in the final model if significant (p<0.05).

For the other long-term health outcomes; adverse reaction to vaccination, growth failure, autoimmune diseases and malignancies; the number of cases was insufficient to perform meaningful multivariable analysis therefore only descriptive statistics are depicted.

A Bonferroni correction was applied to correct for multiple comparisons between outcomes of the three study groups and control group when using multiple pairwise tests. For these analyses, a statistically significant difference was defined as a p<0.02.

All children per mother were included if they fitted the inclusion criteria which may influence outcomes. Therefore, sensitivity analyses were performed by repeating the analyses for only the first born per mother.

### Sample size

The primary outcome that was used for the sample size calculation was the number of antibiotic treated infections during the first 5 years. Based on a previous Dutch study, the rate of infections requiring antibiotic treatment is 43% per year for children between 0 and 4 years.^21^ To detect a difference of 25% in infection rate in children exposed to anti-TNF-α and/or an immunomodulator compared with controls, at a significance level of 0.05 and a power of 80%: 61 children per arm were needed.

### Definitions

Growth failure is defined as abnormal growth resulting in a referral to a paediatrician. Preterm birth is defined as a delivery before 37 weeks of gestation. SGA is a weight below 2 SD for gestational age according to the Dutch reference curve.^22^ The European Surveillance of Congenital Anomalies (EUROCAT) guideline was used to classify congenital abnormalities.^23^ Severe infection is defined as an infection for which hospital admission was necessary.

## Results

**Figure 1:**
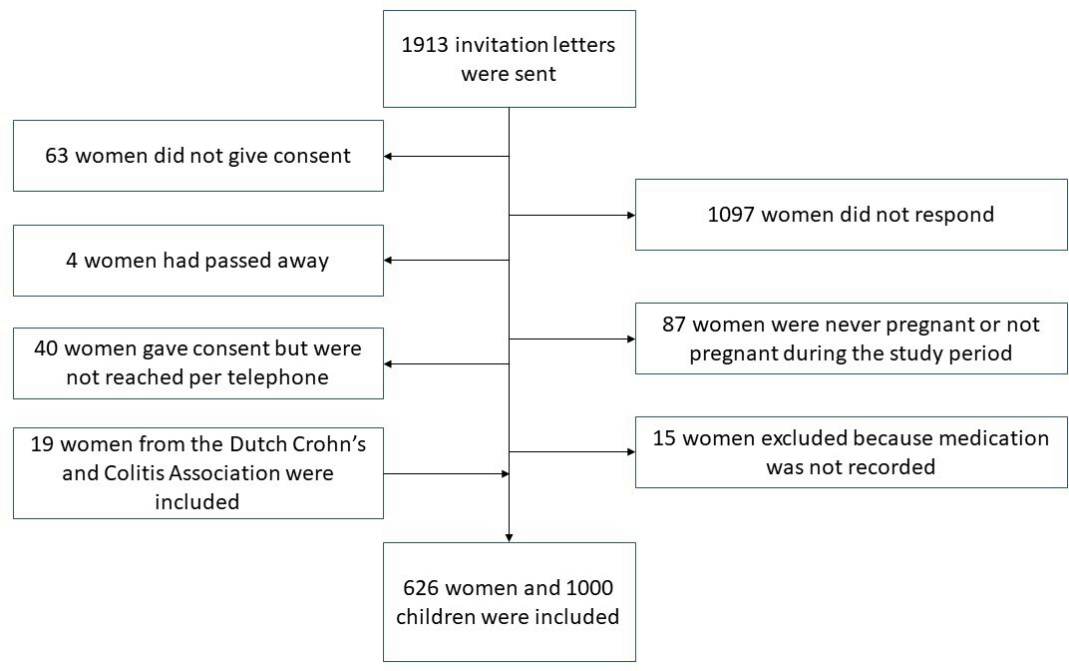
Flow chart of study inclusion.

In total, 20 hospitals participated in this study; 7 university hospitals and 13 general hospitals. A total of 1913 invitation letters were sent. Figure 1 shows a flow chart of the inclusion process. We interviewed the mothers of 1000 children and verified the medical charts of mothers of 855 children (86%) and collected information at GPs from 647 children (65%).

In total, we included 1000 children born to 626 mothers (381 (61%) Crohn’s disease, 225 (36%) ulcerative colitis (UC) and 20 (3%) IBD unclassified). Eight twin pregnancies were included. Overall, 196 (20%) children were exposed to anti-TNF-α in utero (115 infliximab and 81 adalimumab); 86 mothers stopped anti-TNF-α in the third trimester and 110 mothers continued anti-TNF-α during the third trimester. There were 136 (14%) children exposed to anti-TNF-α monotherapy, 240 (24%) children to thiopurine monotherapy and 60 (6%) children to the combination of anti-TNF-α and a thiopurine. Overall, 300 (30%) children were exposed to a thiopurine of who 272 (91%) were exposed during the entire pregnancy, 11 (3%) at conception, 6 (2%) during the first trimester, 8 (3%) during the first and second trimester and 3 (1%) during the second and third trimester. One mother used methotrexate until pregnancy week 6, all others discontinued methotrexate 3–6 months prior to conception. There were 155 children exposed to systemic corticosteroids during pregnancy of who 53 (34%) during the entire pregnancy, 3 (2%) periconceptionally, 12 (9%) during first trimester, 14 (9%) during second trimester, 24 (15%) during third trimester, 5 (3%) during first and second trimester and 20 (13%) during second and third trimester. The trimester of steroid exposure was unknown for 24 (15%) children. Baseline characteristics are shown in table 1.

**Table 1:** Maternal characteristics at the time of childbirth (n=1000)

	Controls (n=564)	Anti-TNF-α monotherapy (n=136)	P value	Thiopurine monotherapy (n=240)	P value	Anti-TNF-α and thiopurine (n=60)	P value
Median maternal age at birth (IQR)	32 (29–35)	30 (28–33)	<0.01	32 (29–35)	0.26	32 (30–34)	0.38
Education level (%)							
High	270 (51)	54 (45)	0.27	111 (50)	0.75	31 (53)	0.68
Secondary	225 (43)	57 (48)	0.36	95 (42)	1	24 (43)	1
Low	34 (6)	9 (7)	0.69	18 (8)	0.43	2 (4)	0.56
Diagnosis (%)							
Crohn’s disease	289 (51)	112 (82)	<0.01	170 (71)	<0.01	42 (70)	<0.01
Ulcerative colitis	252 (45)	22 (16)	<0.01	66 (27)	<0.01	15 (25)	<0.01
IBD unclassified	23 (4)	2 (2)	0.2	5 (2)	0.21	3 (5)	0.73
Disease location CD (montreal) (%)							
L1 Ileal	43 (15)	15 (14)	0.87	20 (12)	0.33	7 (18)	0.64
L2 Colonic	73 (26)	23 (22)	0.43	40 (24)	0.66	7 (18)	0.33
L3 Ileocolonic	170 (59)	69 (64)	0.42	111 (64)	0.28	26 (64)	0.61
Disease behaviour CD (montreal) (%)							
B1 non-stricturing non penetrating	136 (50)	61 (56)	0.37	78 (47)	0.43	16 (40)	0.24
B2 stricturing	48 (18)	9 (8)	0.02	27 (16)	0.7	3 (8)	0.11
B3 penetrating	47 (18)	20 (18)	0.88	40 (25)	0.11	15 (37)	<0.01
B2 + B3 stricturing and penetrating	37 (14)	19 (17)	0.43	22 (12)	0.89	6 (15)	0.81
Perianal fistulising disease CD (%)	67 (24)	35 (32)	0.13	41 (24)	0.91	21 (54)	<0.01
Disease extent UC/IBDU (montreal) (%)							
E1 proctitis	86 (34)	2 (9)	0.02	15 (23)	0.1	2 (12)	0.1
E2 left-sided colitis	69 (27)	6 (26)	1	24 (37)	0.17	3 (19)	0.57
E3 pancolitis	99 (39)	15 (65)	0.03	27 (40)	0.78	11 (69)	0.03
Disease duration in years (IQR)	8 (5–13)	9 (5–12)	0.77	7 (5–12)	0.05	8 (5–12)	0.34
Concomitant IBD medication use (%)							
Systemic steroid (alone or in combination with 5-ASA)	79 (14)	24 (18)	0.28	46 (19)	0.07	6 (10)	0.55
5-ASA (no steroids)	256 (45)	6 (4)	<0.01	55 (23)	<0.01	8 (13)	<0.01
No concomitant IBD medication	229 (41)	106 (78)	<0.01	139 (58)	<0.01	46 (77)	<0.01
IBD abdominal surgery prior to pregnancy (%)	145 (26)	36 (27)	0.91	58 (24)	0.66	10 (17)	0.16
Nulliparous (%)	275 (49)	77 (57)	0.08	132 (55)	0.11	33 (56)	0.34
Folic acid intake (%)	506 (94)	123 (99)	0.01	209 (91)	0.16	49 (93)	0.56

Study groups were compared with the controls. The Bonferroni correction was applied to adjust for multiple testing. A statistically significant difference was defined as a P<0.02.

5-ASA, 5-aminosalicylic acid; CD, Crohn’s disease; IBD, inflammatory bowel disease; IBDU, inflammatory bowel disease unclassified; IQR, interquartile range; TNF, tumour necrosis factor; UC, ulcerative colitis.

### Maternal outcomes

Maternal outcomes are displayed in table 2. Women in the control group more often breastfed than women in the study groups. Women using combination treatment more often had an endoscopy during the pregnancy. We found no other differences between study groups and controls.

**Table 2:** Maternal outcomes (n=1000)

	Controls (n=564)	Anti-TNF-α monotherapy (n=136)	P value	Thiopurine monotherapy (n=240)	P value	Anti-TNF-α and thiopurine (n=60)	P value
Disease activity during pregnancy necessitating medical adjustment (%)	145 (26)	31 (23)	0.51	45 (19)	0.04	14 (24)	0.88
Endoscopy during pregnancy (sigmoidoscopy/colonoscopy) (%)	44 (8)	12 (9)	0.72	15 (6)	0.55	11 (18)	0.01
Anti-TNF cessation in the third trimester (%)	–	66 (49)		–		28 (47)	
Obstetric complications during pregnancy (%)	58 (10)	15 (11)	0.76	32 (13)	0.22	13 (22)	0.02
Smoking during entire pregnancy (%)	24 (4)	12 (9)	0.05	14 (6)	0.37	3 (5)	0.73
Breast feeding >4 weeks (%)	311 (56)	40 (30)	<0.01	50 (22)	<0.01	11 (19)	<0.01

Study groups were compared with the controls. The Bonferroni correction was applied to adjust for multiple testing. A statistically significant difference was defined as a P < 0.02.

TNF, tumour necrosis factor.

#### Obstetric complications

All obstetric complications are displayed in table 3. We found a higher rate of ICP in women using a thiopurine (n=12, 4%) than in women without a thiopurine (n=4, 0.6%), p<0.01. There were no other differences between the study groups and controls.

**Table 3:** Types of obstetric complications (n=1000)

	Controls (n=564)	Anti-TNF-α monotherapy (n=136)	P value	Thiopurine monotherapy (n=240)	P value	Anti-TNF-α and thiopurine (n=60)	P value
Hyperemesis gravidarum (%)	5 (1)	0 (0)	0.59	2 (1)	1.00	0 (0)	1.00
Intrahepatic cholestasis of pregnancy (ICP) (%)	3 (1)	1 (1)	0.58	9 (4)	<0.01	3 (5)	0.01
In utero growth restriction (%)	2 (1)	2 (1)	0.17	2 (1)	0.59	0 (0)	1.00
Hypertension/pre-eclampsia (%)	28 (5)	6 (4)	1.00	13 (5)	0.86	7 (13)	0.07
HELLP (%)	4 (1)	1 (1)	1.00	2 (1)	1.00	2 (3)	0.11
Gestational diabetes (%)	16 (3)	5 (3)	0.58	4 (1)	0.46	1 (1)	1.00

Study groups were compared with the controls. The Bonferroni correction was applied to adjust for multiple testing. A statistically significant difference was defined as a P < 0.02.

HELLP, haemolysis, elevated liver enzyme levels and low platelet levels; TNF, tumour necrosis factor.

### Birth outcomes

Birth outcomes are displayed in table 4. Women using anti-TNF-α monotherapy more often had a caesarean section than controls. No other differences were found.

In total 291 (29%) caesarean sections were reported. The indication for a caesarean section was related to the underlying IBD in 107 cases (37%) namely; perianal fistulising disease (n=86), ileo pouch anal anastomosis (n=16), disease activity (n=3) and stoma (n=2).

**Table 4:** Birth outcomes (n=1000)

	Controls (n=564)	Anti-TNF-α monotherapy (n=163)	P value	Thiopurine monotherapy (n=240)	P value	Anti-TNF-α and thiopurine (n=60)	P value
Birth weight in kg (IQR)	3.3 (3.0–3.7)	3.3 (2.9–3.7)	0.97	3.3 (3.0–3.6)	0.68	3.2 (2.7–3.5)	0.39
Gestational age in weeks (IQR)	39 (38–40)	39 (38–40)	0.55	39 (38–40)	0.07	39 (37–40)	0.09
Small for gestational age (%)	22 (4)	4 (3)	0.80	6 (3)	0.40	2 (3)	1.00
Preterm birth (%)	61 (11)	14 (10)	1.00	33 (14)	0.28	10 (17)	0.20
Major congenital abnormalities (%)	10 (2)	6 (4)	0.10	8 (3)	0.20	3 (5)	0.12
Caesarean section (%)	142 (25)	56 (42)	<0.01	75 (32)	0.06	18 (31)	0.35

Study groups were compared with controls. The Bonferroni correction was applied to adjust for multiple testing. A statistically significant difference was defined as a P < 0.02.

IQR, interquartile range; TNF, tumour necrosis factor.

Major congenital abnormalities were seen in 27 (2.7%) children. Univariable logistic regression analysis showed no association between major congenital abnormalities and the treatment groups anti-TNF-α monotherapy (OR 1.85, 95% CI 0.73 to 4.67, p value 0.19), thiopurine monotherapy (OR 1.34, 95% CI 0.58 to 3.11, p value 0.49) and combination treatment of anti-TNF-α & thiopurine (OR 2.25, 95% CI 0.59 to 6.86, p= 0.27). There was an insufficient number of cases with congenital abnormalities to allow reliable estimation of a multivariable model.

In total, 118 (12%) children were born preterm. In the univariable logistic regression analysis we found an association with systemic corticosteroid use (p<0.01), obstetrical complications (p<0.01), disease activity (p=0.02) and endoscopy during pregnancy (p=0.02) (table 5). In a multivariable analysis, we included the latter variables and the treatment groups. This model estimated an association between the variables systemic corticosteroid use and obstetric complications and the outcome preterm birth, however, anti-TNF-α and/or thiopurine use was not associated with preterm birth.

**Table 5:** Multivariable logistic regression model for preterm birth (n=1000)

	Univariable analyses			Multivariable analyses		
	OR	95% CI	P value	aOR	95% CI	P value
Anti-TNF-α monotherapy	0.84	0.46 to 1.51	0.55	0.84	0.44 to 1.26	0.61
Thiopurine monotherapy	1.26	0.82 to 1.94	0.29	1.16	0.71 to 1.88	0.55
Anti-TNF-α and thiopurine	1.54	0.76 to 3.12	0.23	1.43	0.66 to 3.12	0.37
Systemic corticosteroids	2.81	1.81 to 4.35	<0.01	2.62	1.56 to 4.38	<0.01
Disease activity	1.61	1.06 to 2.46	0.02	1.06	0.61 to 1.89	1.83
IBD type (CD or UC/IBDU)	0.81	0.57 to 1.17	0.26			
Maternal age	0.97	0.93 to 1.02	0.28			
Smoking during pregnancy	0.98	0.41 to 2.35	0.96			
Obstetric complications	1.35	1.22 to 1.49	<0.01	4.07	2.54 to 6.54	<0.01
Endoscopy during pregnancy	1.94	1.08 to 3.47	0.02	1.17	0.57 to 2.38	0.83

aOR, adjusted odds ratio; CD, Crohn’s disease; IBD, inflammatory bowel disease; IBDU, inflammatory bowel disease unclassified; TNF, tumour necrosis factor; UC, ulcerative colitis.

A total of 34 (3%) children were born SGA. In a univariable logistic regression analysis we found no association between SGA and the treatment groups anti-TNF-α monotherapy (OR 0.84, 95% CI 0.29 to 2.41, p=0.74), thiopurine monotherapy (OR 0.67, 95% CI 0.27 to 1.63, p=0.37) and combination treatment of anti-TNF-α and thiopurine (OR 0.97, 95% CI 0.23 to 4.17, p=0.97). There was an insufficient number of cases with abnormalities to allow reliable estimation of a multivariable model.

#### Subanalysis for children born to mothers with ICP

There were 16 children born to mothers with ICP, of whom 12 (75%) used thiopurine during pregnancy. None of the children born to mothers with ICP had a congenital abnormality or were born SGA. Women with ICP gave birth preterm more often (n=8, 38%) than women without ICP (n=109, 11%) (p<0.01) and women with ICP had a caesarean section (n=9, 56%) more often than women without ICP (n=283, 29%) (p=0.03).

### Long-term health outcomes of children

Median total follow-up time was 60 (IQR 24–60) months. Follow-up time was shorter for children in the anti-TNF-α monotherapy group (24 (IQR 12–48) months) (p<0.01), the thiopurine monotherapy group (60 (IQR 36–60) months) (p<0.01) and the combination treatment group (36 (IQR 12–60) months) (p<0.01) compared with controls (60 (IQR 48–60) months).

The sensitivity and specificity of mother-reported antibiotic treated infections in the first year of life was 53.8% (95% CI 45.3% to 62.2%) and 89.4% (95% CI 85.0% to 92.6%), respectively. Therefore, for the outcome antibiotic-treated infections we only used GP reported information (n=647). For all other long-term health outcomes there was no significant difference in reporting between mothers and GPs.

In our overall study group most children (85%) attended day care. Median age at which the child first attended daycare was 8 (IQR 3–24) months. Fifty-three per cent of the children attended day care before their first birthday.

#### Antibiotic-treated infections

There were 444 antibiotic courses reported per 1000 person-years. The following infections were most often reported: 502 (42%) ear, nose and throat (ENT) related infections and 441 (37%) respiratory tract infections.

In the univariable analyses, we found a statistically significant association between the outcome antibiotic-treated infections and the variable smoking during pregnancy (table 6). In the multivariable analysis, we included the following variables: treatment groups, smoking during pregnancy, obstetric complications and breast feeding. An interaction term was added to the model because of a statistically significant association between the variables breastfeeding and treatment groups. This multivariable model shows an increased rate of antibiotic-treated infections in offspring in the cases mothers actively smoked during pregnancy and a decreased rate of antibiotic treated infections in case mothers breastfed for at least 1 month. In utero exposure to IBD medication was, however, not associated with antibiotic-treated infections.

**Table 6:** Multivariable negative binomial regression model for antibiotic-treated infections (n=647)

	Univariable analyses			Multivariable analysis		
	IRR	95% CI	P value	aIRR	95% CI	P value
Anti-TNF-α monotherapy	1.25	0.94 to 1.68	0.83	0.66	0.45 to 0.98	0.04
Thiopurine monotherapy	1.10	0.87 to 1.40	0.41	1.04	0.78 to 1.38	0.81
Anti-TNF-α and thiopurine	1.05	0.69 to 1.60	0.13	0.74	0.45 to 1.22	0.24
Systemic corticosteroids	1.09	0.85 to 1.40	0.49			
ICP	1.60	0.82 to 3.13	0.17			
Smoking during pregnancy	2.15	1.38 to 3.34	<0.01	1.90	1.19 to 3.04	<0.01
Disease activity	1.08	0.86 to 1.37	0.49			
Obstetric complications	1.31	0.99 to 1.73	0.06	1.31	0.99 to 1.75	0.06
Preterm birth	1.23	0.94 to 1.61	0.14			
Daycare attendance	0.89	0.66 to 1.21	0.47			
Breast feeding (>1 month)	0.84	0.69 to 1.02	0.08	0.70	0.54 to 0.90	<0.01

aIRR, Adjusted Incidence Rate Ratio; ICP, intrahepatic cholestasis of pregnancy; IRR, incidence rate ratio; TNF, tumour necrosis factor.

##### Subgroup anti-TNF-α exposed children

Information from GP’s regarding antibiotic-treated infections was retrieved from 140 children who were exposed to anti-TNF-α in utero. Within this anti-TNF-α exposed group there were 79 (56%) exposed in the third trimester of pregnancy. In a univariable negative binomial regression analysis, we found no association between continuing anti-TNF-α in the third trimester of pregnancy and the number of antibiotic-treated infections (IRR 1.06, 95% CI 0.68 to 1.66, p=0.78).

#### Hospital admission due to a severe infection

In total, 107 (11%) children were admitted to hospital because of a severe infection of who six were admitted twice. Hospital admission per treatment group was as follows: 13 (10%) in the anti-TNF-α monotherapy group, 30 (13%) in the thiopurine monotherapy group, 6 (10%) in the combination group and 58 (10%) in the control group. Median age during first hospital admission was 6 (IQR 6–18) months. Hospital admission occurred most often in the first year of life (n=76, 69%).

The reasons for hospital admission were mostly an acute respiratory tract infection (n=37, 31%), viral infections (n=27, 23%), fever (n=15, 13%), urinary tract infection (n=10, 8%) or sepsis (n=9, 8%).

In the univariable analysis, we found no statistical significant association between any of the candidate predictor variables and the outcome severe infection (table 7). The predictor variable ICP was not assessed because none of the children born to mothers with ICP had a severe infection. In the multivariable analysis, we included the variables; treatment groups and the use of systemic corticosteroids. The final model showed no association between in utero exposure to IBD treatment and severe infections in offspring.

**Table 7:** Multivariable negative binomial regression model for hospital admission (n=1000)

	Univariable analyses			Multivariable analysis		
	IRR	95% CI	P value	aIRR	95% CI	P value
Anti-TNF-α monotherapy	1.67	0.92 to 3.06	0.10	1.66	0.91 to 3.04	0.10
Thiopurine monotherapy	1.40	0.88 to 2.22	0.15	1.35	0.85 to 2.15	0.20
Anti-TNF-α and thiopurine	1.63	0.71 to 3.78	0.25	1.65	0.71 to 3.82	0.24
Systemic corticosteroids	1.55	0.97 to 2.50	0.07	1.52	0.94 to 2.44	0.09
Smoking during pregnancy	1.52	0.70 to 3.32	0.29			
Disease activity	1.27	0.81 to 1.98	0.30			
Obstetric complications	0.92	0.49 to 1.74	0.80			
Preterm birth	1.17	0.66 to 2.05	0.60			
Day care attendance	1.14	0.60 to 2.15	0.69			
Breast feeding (>1 month)	0.95	0.64 to 1.40	0.79			

aIRR, adjusted incidence rate ratio; IRR, incidence rate ratio; TNF, tumour necrosis factor.

##### Subgroup anti-TNF-α exposed children

There were 196 children exposed to anti-TNF-α during the pregnancy of which 110 (56%) were exposed in the third trimester, others were only exposed in the first and/or second trimester. In a negative binomial regression analysis, we found no association between continuing anti-TNF-α in the third trimester of pregnancy and severe infections in offspring (IRR 0.46, 95% CI 0.16 to 1.32, p=0.15).

#### Sensitivity analyses

For the sensitivity analyses, we included only the first born per mother (n=626). Excluding siblings from analyses did not affect the long-term health outcomes.

#### Adverse reaction to vaccination

In total, seven adverse reactions to vaccinations were reported; four children were admitted to hospital because of a high fever, two children received antibiotic treatment because of an infection and one child received antihistamine because of severe erythema at the injection site. No life-threatening reactions were reported. The mothers of these children used the following IBD treatment during pregnancy: thiopurine monotherapy (n=2), anti-TNF-α and thiopurine (n=1), no anti-TNF-α or thiopurine (n=4). None of the children born to mothers with ICP had a an adverse reactions to a vaccination.

Overall, maternal IBD treatment does not seem to be associated with adverse reactions to vaccinations.

#### Growth

In total, there were 16 children with growth failure. All children with growth failure were referred to a paediatrician for further analysis. Mothers used the following IBD treatment during pregnancy: anti-TNF-α monotherapy (n=4), thiopurine monotherapy (n=3), no anti-TNF-α or thiopurine (n=9). None of the children born to mothers with ICP had a growth failure. We found no association between maternal IBD treatment during pregnancy and growth failure.

#### Autoimmune diseases and malignancies

There was 1 (0.1%) child diagnosed with an autoimmune disease: diabetes mellitus type 1 at the age of 4. This child was born to a mother with UC who did neither use IBD medication during pregnancy nor experienced disease activity during the pregnancy.

There were 2 (0.2%) children diagnosed with a malignancy; a rhabdomyosarcoma of the left orbita at the age of 3 and leukaemia at the age of 2. Both mothers were diagnosed with UC. Both mothers used corticosteroids during the entire pregnancy and the mother of the child with a rhabdomyosarcoma used azathioprine during the entire pregnancy. None of the children born to mothers with ICP had an auto-immune disease or malignancy. Overall, autoimmune diseases and malignancies does not seem to be associated with maternal IBD treatment during pregnancy.

## Discussion

In this large multicentre retrospective study, we found no association between maternal anti-TNF-α and/or thiopurine use for IBD during pregnancy and adverse long-term health outcomes of their offspring until 5 years of age.

Our study shows that in utero exposure to anti-TNF-α and/or thiopurine does not increase the risk of an antibiotic-treated infection or hospital admission because of a severe infection in children during their first 5 years of life. However, smoking during pregnancy increased the risk of an antibiotic-treated infection in offspring and breastfeeding for at least 1 month decreased the risk of an antibiotic-treated infection in offspring. The rate of antibiotic-treated infections is slightly lower in the anti-TNF-α monotherapy group compared with controls, which is possibly a result of the counselling that mothers receive in which they are advised to avoid infectious sources as anti-TNF-α is actively transported over the placenta to the newborn in the second but mostly the third trimester. Anti-TNF-α cessation before the third trimester did not influence infection risk, however, this outcome may also be biased by parental counselling. The use of systemic corticosteroids was associated with preterm birth but not with infections in offspring, however, it should be mentioned that systemic corticosteroids were used during different part of the pregnancy. Numbers per trimester were to small for meaningful additional sub-analyses and therefore we were unable to assess the effect of systemic corticosteroids during the different trimesters.

The number of prescribed antibiotic courses is in line with the results of a national survey of Dutch general practices for children between 0 and 4 years.^21^ The most frequent indication for antibiotics in our study were ENT and respiratory tract infections, which is comparable with children of the same age in the general Dutch population.^24–26^ The incidence of severe infections coincides with the results of a Belgian population study.^27^

We found an increased risk of ICP in women using a thiopurine during pregnancy. This association has not been described previously. The pathogenesis of ICP is multifactorial, possibly including hormonal, environmental, genetic and dietary influences but also azathioprine and 6-MP are both associated with liver enzyme abnormalities^28^ including cholestasis.^29^ Pregnancy has an important effect on maternal thiopurine metabolism leading to decreased 6- thioguaninenucleotides (6-TGN) and increased 6-MMP concentrations.^8^ This finding underlines the importance of introducing therapeutic drug monitoring during pregnancy to avoid maternal exposure to high levels of 6-MMP. However, we found no evidence that maternal thiopurine use influences long-term health outcomes of exposed children. But the small sample size should be considered when interpreting this outcome.

In our study, 27 infants (2.7%) were born with a major congenital abnormality, which is consistent with data reported from the overall European population.^30^ Anti-TNF-α and/or thiopurine use for IBD during pregnancy was not associated with the outcome major congenital abnormalities of their offspring. In addition, we also found no association between anti-TNF-α and/or thiopurine use during pregnancy and the outcomes preterm birth and SGA. Unfortunately, the group sizes for the birth outcomes; major congenital abnormalities and SGA, were so small that it was not possible to obtain reliable estimates for adjusted ORs for these analyses and therefore only unadjusted ORs were provided. Unadjusted ORs are more at risk to bias than adjusted ORs due to confounding, however, in the absence of the option to provide adjusted ORs we reported the unadjusted ORs in these cases.

No life-threatening allergic reactions to vaccines occurred in our study. Life-threatening allergic reactions to vaccinations, however, occur very rarely, approximately 1 in 1 000 000 doses,^31^ and therefore, our study group is too small to draw firm conclusions.

Growth failure occurred in 1.5% of cases in our study which was comparable to a Dutch non-IBD control group.^18^

There seems to be no association between maternal IBD treatment and autoimmune diseases or malignancies in offspring. The incidence of a malignancy in the age group 0–5 years in the Netherlands is similar to our study group (0.2%).^32^

Although our study is a large nationwide multicentre study, it was limited by its retrospective design. To address this limitation, information from medical charts of the mothers was collected in each hospital and long-term health outcomes of children were collected from GPs. In a small part of the cohort (14%) medical charts from mothers could not be verified because mothers were not yet a patient at that particular hospital at the time of pregnancy and in some cases information could not be retraced because of bankruptcy of one hospital. Information of children was collected at their GP with consent of both parents and was missing if only one parent gave consent, which occurred more often in the case parents had split up. In addition, our study was subject to response bias as only 34% of the invited women participated in the study. Possibly most invited women did not fit the inclusion criteria and therefore did not respond, however, this was not assessed. Furthermore, our study lacks a non-IBD control group. For each health outcome a literature search was conducted to compare our study group to the general population.

Our study does have advantages over previous published studies. This is the largest long-term study to assess implications of maternal IBD medication during pregnancy on multiple health outcomes of children. In addition, the type of infections per follow-up year was retrieved from medical records of the GP. Therefore, infection rate per age group and type of infection per year could be compared with overall Dutch population. The type of infections necessitating hospital admission was also retrieved. Hospital admission for other indications could therefore be easily excluded. Finally, this is a national study and as a result GPs and paediatricians work according to the same protocols. Differences in antibiotic prescribing and hospital admission are therefore not expected between different regions.

## Conclusion

In our multicentre retrospective study assessing long-term health outcomes of 1000 children born to mothers with IBD, we found that anti-TNF-α and/or thiopurine use during pregnancy does not affect birth outcomes and the following long-term health outcomes of children: antibiotic-treated infections, severe infections needing hospital admission, adverse reactions to vaccinations, growth failure, autoimmune diseases and malignancies. We, however, did find an association between thiopurine use during pregnancy and intrahepatic cholestasis of pregnancy, without affecting birth outcomes and long-term health outcomes.

## Data Availability

Data are available on reasonable request.
